# Supplementary figures and images for: Identification of Male Sex-Related Genes Regulated by SDHB in Macrobrachium nipponense Based on Transcriptome Analysis after an RNAi Knockdown
Source: Int J Mol Sci. 2023 Aug 24;24(17):13176. doi: 10.3390/ijms241713176 (PMC10487615; doi:10.3390/ijms241713176)

# Violin plot for FPKM Values

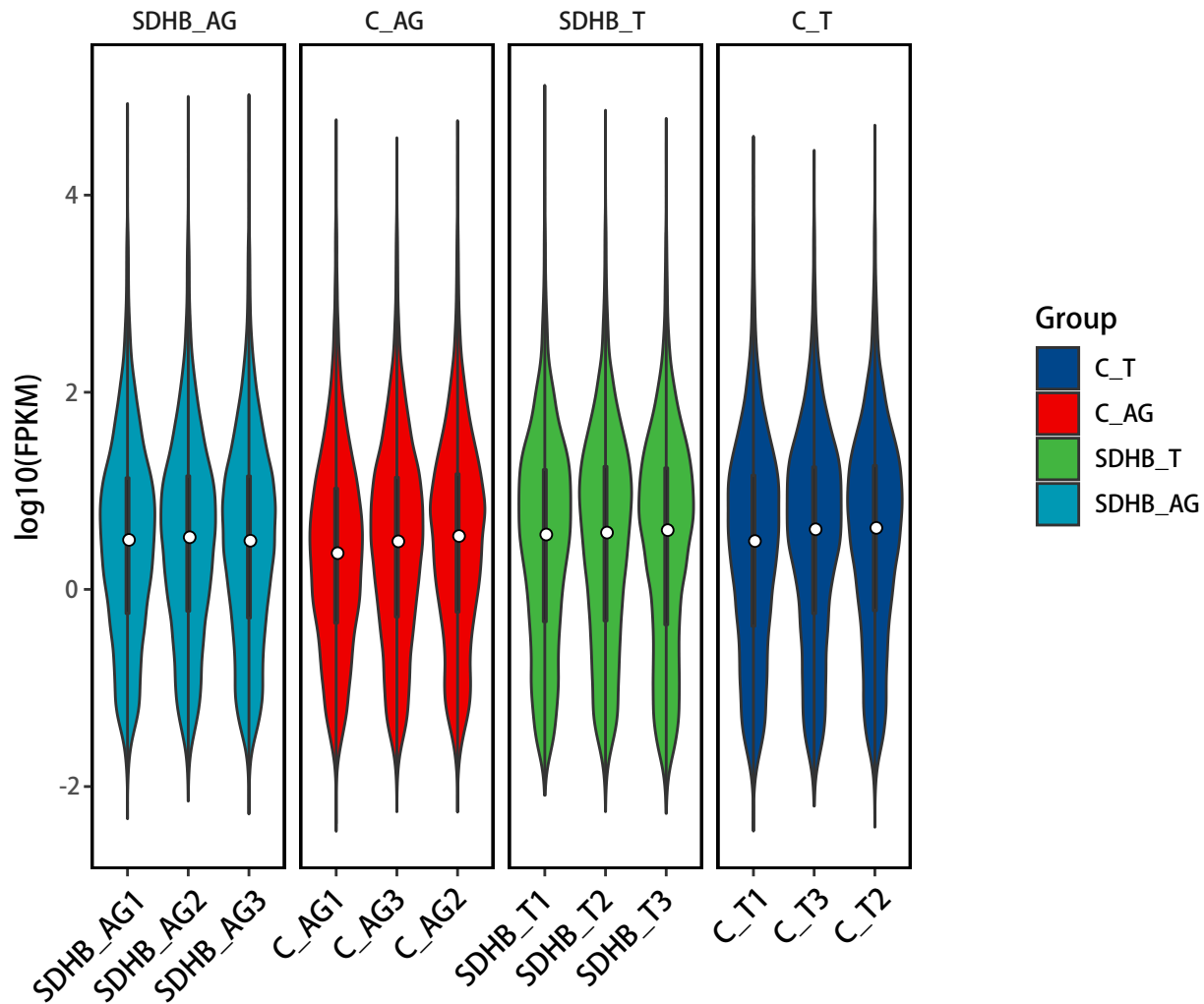

Supplement: Supplementary file 1 [file ijms-24-13176-s001.zip › Figure S1. violin chart for FPKM in 4 groups.pdf]
